# A Case of Granulomatosis with Polyangiitis Causing Hydroureter and Hydronephrosis

**DOI:** 10.1155/2014/713957

**Published:** 2014-01-02

**Authors:** Farzin Farpour, Adriana Abrudescu

**Affiliations:** ^1^Internal Medicine, Mount Sinai School of Medicine, Queens Hospital Center, Jamaica, NY 11432, USA; ^2^Mount Sinai School of Medicine, Queens Hospital Center, Jamaica, NY 11432, USA

## Abstract

Granulomatosis with Polyangiitis (GPA, formerly known as Wegener) is a systemic vasculitis characterized by granulomatous involving upper and lower respiratory tract and can also cause necrotizing glomerulonephritis (Umemoto et al. 2012 and Takala et al. 2011). GPA is associated with antineutrophil cytoplasmic autoantibodies (ANCA) against serine proteinase 3 (PR3) (Takala et al. 2011, Dufour et al. 2012, and Berthoux et al. 2011). This disease usually starts with involvement of the upper and lower respiratory tracts and also can involve kidney, eyes, skin, central and peripheral nervous systems, and gastrointestinal tract (Umemoto et al. 2012, Takala et al. 2011, and Berthoux et al. 2011). We describe a case of GPA that presented with abdominal pain. Computed tomography (CT) scan with contrast showed right sided moderate hydronephrosis and hydroureter, to the level of the right common iliac artery. There was also mural segmental thickening in common iliac artery which was thought to be the cause of the ureteral obstruction and hydronephrosis. Our case shows that mural segmental thickening in common iliac artery happened due to GPA and caused hydronephrosis. In addition, most of the cases with hydronephrosis due to GPA went through urology intervention such as stent placement but in our case hydronephrosis resolved with medical management.

## 1. Introduction 

GPA is a systemic vasculitis characterized by changes involving upper and lower respiratory tracts and can also cause necrotizing glomerulonephritis [1, 2].

GPA is associated with an antineutrophil cytoplasmic autoantibodies (ANCA) against serine proteinase 3 (PR3) [2–4].

This disease usually starts with the involvement of the upper and lower respiratory tracts and also can involve kidney, eyes, skin, central and peripheral nervous systems, and gastrointestinal tract [1, 2, 4].

Usually the small vessels are the primary target of GPA; however, several recent reports have shown that the large vessels such as aorta and its branches can be involved.

GPA can affect any organ, however, urologic involvement has rarely been reported.

Here, we report a case of GPA presented with right kidney hydronephrosis. CT studies showed mural segmental thickening in common iliac artery, which was the cause of the ureteral obstruction and hydronephrosis.

## 2. Case Report

A 44-year-old man was admitted to our hospital with a one-day history of sever right lower quadrant abdominal pain. He described the pain as persistent and very severe, 10/10, radiating to the flank associated with nausea and fivetime vomiting. He denied any urinary complains or fever. His past medical history was significant for PGA which was diagnosed two years prior to admission. Biopsy at that time showed necrotizing granulomatous vasculitis. However, he didi nit complain of Methotrexate and prednisone. His other medical history was remarkable for hypertension.

Physical examination was significant for severe tenderness in the lower right lower quadrant of abdomen and also presence of saddle nose deformity.

Laboratory workup was significant for leukocyte count of 12,300/mL, platelet count of 318,000/lL, C-reactive protein (CRP) level 5.4 mg/dL and ESR level 45 mm/hr, serum creatinine level 1.1 mg/dL, positive ANCA with specificity for anti-PR3 level 65 U/mL, C3 and C4 normal, and ANA negative. Urinalysis showed normal sediment and no proteinuria.

**Figure 1:**
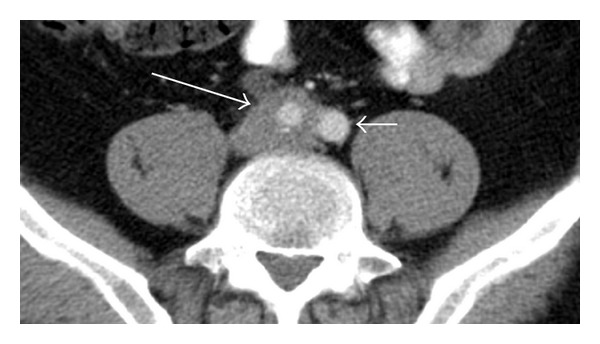
CAT scan axial view shows mural segmental thickening in common iliac artery (large arrow) and hydroureter (small arrow).

**Figure 2:**
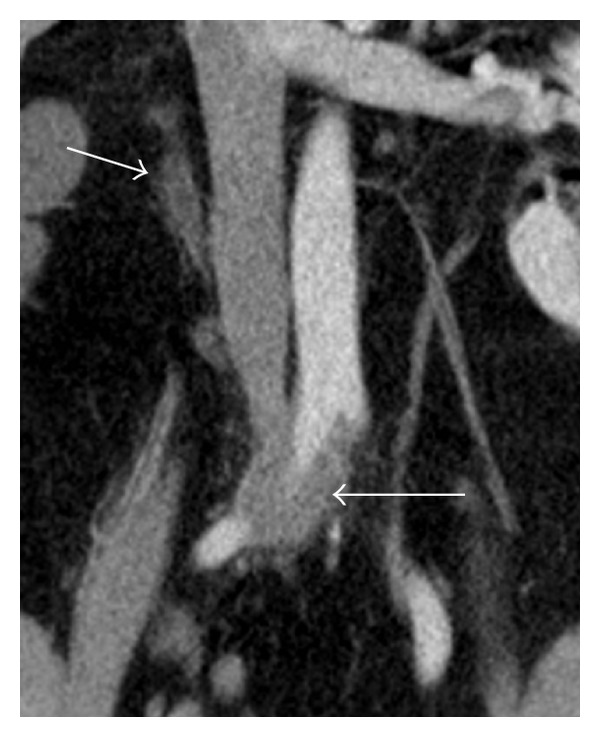
CAT scan coronal view shows mural segmental thickening in common iliac artery (large arrow) and hydroureter (small arrow).

**Figure 3:**
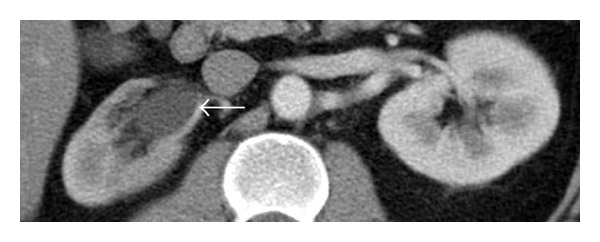
CAT scan axial view shows hydronephrosis (arrow) in right kidney.

CAT scan of abdomen showed right sided moderate hydronephrosis and hydroureter, to the level of the right common iliac artery. But no stone was noted. There was also mural segmental thickening in common iliac artery (Figures 1, 2, and 3).

**Figure 4:**
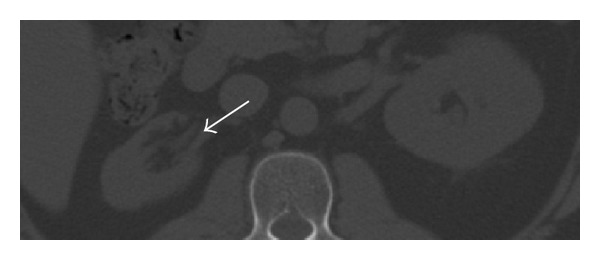
CAT scan axial view showed resolved hydronephrosis (arrow) in right kidney.

Because of prior history of GPA and CAT scan finding and high ESR and CRP and positive ANCA, the clinical diagnosis was in favor of active PGA. Therefore, he was started on high dose methylprednisolone of 1 mg/kg intravenous. Initially he was scheduled for urology intervention; however since pain was gone and CAT scan showed resolved of hydronephrosis (Figure 4), intervention was cancelled.

Repeat laboratory showed C-reactive protein (CRP) level 1 mg/dL, ESR level 10 mm/hr, and serum creatinine level 0.7 mg/dL.

Methylprednisolone was changed to oral prednisone and Methotrexate 15 mg/week was also added to management. Prednisone was gradually tapered down. He was discharged home since presenting symptom resolved to continue low dose prednisone. Since he remained asymptomatic was discharged home.

He was followed for 2 years in clinic and no abdominal pain or hydronephrosis was noted.

## 3. Discussion

GPA is a systemic necrotizing vasculitis that usually involves respiratory tract, lungs, and urologic system. Renal involvement is considered as the most urologic manifestation of GPA. Renal involvement usually presents as rapidly progressive segmental glomerulonephritis with proteinuria. Renal mass also was reported due to GPA [5, 6].

Ureters rarely can be involved due to GPA. To our knowledge only six documented cases were found in review of the English literature regarding ureteral stenosis secondary to GPA [1, 3, 6–8].

Ureters usually involve in GPA like our case in which ureteral stenosis was in the level of iliac artery, however, multilevel or bilateral lesions have also been reported [3, 6].

Bilateral compression of ureter can cause acute renal failure with anuria. However, in this case there was no anuria or renal failure since it was unilateral ureter involvement.

Ureteral stenosis is rarely reported as a recurrent feature of GPA [3], such as our case in which no recurrence was noted so far.

Le Thi Huong et al. reported 80 cases with urogenital involvement due to GPA in which only 2 cases with ureteral stenosis were found and only one of cases had bilateral ureteral stenosis. Ureteral double-J stents were inserted for bilateral ureteral obstruction. The treatment was followed by high dose corticosteroid and cyclophosphamide and the entire urinary symptom resolved [6].

Umemoto et al. reported a case of abdominal pain with ureteral obstruction, hydronephrosis, and perinephric urinary leakage due to inflammation around the iliac artery due to GPA. Initially it was presumed that the abdominal pain is due to infection and Methotrexate was stopped and antibiotic was started, however abdominal pain was getting worse and CT showed hydronephrosis. A double-J stent was inserted into the left ureter. Management was followed by methylprednisolone pulse therapy and intravenous cyclophosphamide pulse therapy and oral prednisone 40 mg daily and gradually tapered down to low dose. Repeat imaging study showed complete resolve of the lesion. After 6 months, monthly intravenous cyclophosphamide was switched to weekly Methotrexate. The patient remained asymptomatic [1].

Lillaz et al. reported one case of ureter obstruction due to GPA. In this case the obstruction was initially resolved with stent placement followed by steroid and cyclophosphamide therapies. Her renal function and global status improved immediately [7].

Dufour et al. evaluated 11 patients with urogenital manifestation of GPA but only one patient had ureteral obstruction which was on prednisone and Methotrexate. Initially ureteral catheter was inserted. However after 6 months resection of the stricture with end-to-end anastomosis was done due to persistence of the ureteral obstruction. Immunosuppressive therapy was not changed and no obstruction was noted after 3 years [3].

Davenport et al. reported eight PGA cases with urogenital involvement but only one case with ureter obstruction was found which was due to necrotic debris at the bladder base and at both ureteric orifices. He was started on 10 immunosuppressive therapy but unfortunately he died of sepsis [8].

Ureteral stenosis has also been reported in other forms of small to medium-sized vessel vasculitis, such as polyarteritis nodosa and Henoch Schonlein purpura [1]. However, to our knowledge, this is the second case in which inflammation of the peri-iliac artery causes ureteral obstruction and hydronephrosis due to GPA.

In most of these cases, unfortunately immunosuppressive management was not started right after diagnosis of ureteral obstruction, but instead surgery was done. However, in most of the cases management was followed by combined glucocorticoid and cyclophosphamide therapy and then switched to a less toxic immunosuppressant, such as Methotrexate. In most of the cases obstruction was completely resolved after immunosuppressive therapy except one case that remained unknown since the patient died of sepsis. There was only one case that ended up with surgery while on immunosuppressive therapy: however, in that one case no change in immunosuppressive therapy was done prior to surgery. Surgery could have been possibly avoided by a change in immunosuppressive management [1, 3, 5–8].

In summary, we presented a case of GPA with peri-iliac arterial inflammation that led to ureteral obstruction and hydronephrosis, and obstruction was resolved with immunosuppressive therapy. It seems logical to consider medical management as a fist line therapy in any urologic involvement of GPA. However, in patients with persistent symptoms surgical procedures, such as stent placement, should be regarded. However, further investigations are required to confirm this consideration.
